# On the Role of Central Type-1 Cannabinoid Receptor Gene Regulation in Food Intake and Eating Behaviors

**DOI:** 10.3390/ijms22010398

**Published:** 2021-01-01

**Authors:** Mariangela Pucci, Elizabeta Zaplatic, Maria Vittoria Micioni Di Bonaventura, Emanuela Micioni Di Bonaventura, Paolo De Cristofaro, Mauro Maccarrone, Carlo Cifani, Claudio D’Addario

**Affiliations:** 1 Faculty of Bioscience and Technology for Food, Agriculture and Environment, University of Teramo, 64100 Teramo TE, Italy; ezaplatic@unite.it; 2 Department of Biosciences and Nutrition, Karolinska Institutet, 14183 Huddinge, Sweden; 3 Pharmacology Unit, School of Pharmacy, University of Camerino, 62032 Camerino MC, Italy; mariavittoria.micioni@unicam.it (M.V.M.D.B.); emanuela.micioni@unicam.it (E.M.D.B.); carlo.cifani@unicam.it (C.C.); 4 Clinical Endocrinology and Metabolism Outpatient Clinic, 64021 Giulianova TE, Italy; pdecristofaro@gmail.com; 5 Department of Biotechnological and Applied Clinical Sciences, University of L’Aquila, 67100 L’Aquila AQ, Italy; mauro.maccarrone@univaq.it; 6 European Center for Brain Research, Santa Lucia Foundation IRCCS, 00179 Rome RM, Italy; 7 Department of Clinical Neuroscience, Karolinska Institutet, 17176 Stockholm, Sweden

**Keywords:** type-1 cannabinoid receptor gene, transcriptional regulation, food intake, eating behaviors

## Abstract

Different neuromodulatory systems are involved in long-term energy balance and body weight and, among these, evidence shows that the endocannabinoid system, in particular the activation of type-1 cannabinoid receptor, plays a key role. We here review current literature focusing on the role of the gene encoding type-1 cannabinoid receptors in the CNS and on the modulation of its expression by food intake and specific eating behaviors. We point out the importance to further investigate how environmental cues might have a role in the development of obesity as well as eating disorders through the transcriptional regulation of this gene in order to prevent or to treat these pathologies.

## 1. Central Regulation of Food Intake and the Role of Cannabinoid Receptor Type-1

Food intake might be considered the integration of humoral and neuronal signals processed by the nervous system for the balance of energy and of sensory cues, as well as of the motivational and emotional state of an individual. Thus, different eating behaviors are finely driven by both homeostatic and hedonic signals, whose functions may vary between individuals according to previous experiences and/or epigenetic variations [1,2,3,4,5,6,7].

Homeostatic and hedonic central circuitries are interconnected, in fact feeding behaviors are affected by brain regions classically viewed as mainly involved in homeostatic feeding; however, these are also influenced by brain corticolimbic and hedonic areas, and vice versa [8,9]. The homeostatic feeding will be terminated once the organism is repleted with energy and nutrients, while hedonic feeding might continue. An imbalance toward the hedonic aspect of feeding without restriction may provoke changes in the food intake with serious consequences on the weight gain/loss [10,11].

The hypothalamus (HYP) is the center for the integration and control of essential bodily functions, such as circadian rhythm, body temperature and plasma-osmolarity, and traditionally recognized as the main brain region regulating food intake. It regulates feeding as a function of caloric and nutritional requirements, by sensing macronutrients and through the action of circulating regulatory hormones, neuropeptides and neuromodulators, such as leptin, cholecystokinin, ghrelin, orexin/hypocretin, insulin, neuropeptide Y, and notably lipid signals like endocannabinoids [12,13,14,15]. The imbalance in hypothalamic function may provoke an altered food intake, potentially leading to eating disorders (EDs) and obesity [16,17,18].

Besides HYP, several limbic brain areas including ventral tegmental area, nucleus accumbens (NAc), amygdala, and hippocampus, as well as cortical brain regions, have also been implicated in the hedonic aspects of feeding [19,20,21].

Studies on the role played by the reward circuits in defining hedonic aspects of feeding allowed to define how common mechanisms are shared by drug abuse and food addiction [22,23,24]. Both are compulsive behavioral disorders that induce alterations in brain mechanisms underlying synaptic plasticity and energy homeostasis, showing common vulnerabilities and pathophysiological aspects [25].

Among the different neuromodulatory systems involved in long-term energy balance and body weight regulation, many preclinical and clinical evidence show the key role of the endocannabinoid system (ECS) [26], and in particular, the activation of type-1 cannabinoid receptors (CB_1_R) [27,28].

Indeed, several preclinical studies show that orexigenic stimuli induce CB_1_R activation in the rat brain, specifically in the HYP [29,30] where CB_1_R positive neurons are present in different nuclei [31], although at low density [32], and support a role in food and energy balance [33,34,35,36]. Brain reward pathways are largely responsible for processing information related to the motivation, expectation, and pursuit of pleasurable experiences, and CB_1_R signaling was reported to modulate dopaminergic signaling in the ventral tegmental area and NAc to control hedonic eating [37,38,39,40]. CB_1_R signaling also plays a role in the functional activity of caudal brainstem nuclei: parabrachial nucleus, nucleus of the solitary tract, and dorsal motor nucleus of the vagus nerve. Herein, CB_1_R mainly controls food preferences, e.g., digestion of fat rich palatable food [37]. Several experimental findings already pointed to CB_1_R as therapeutic target to treat altered feeding behavior and obesity [30,34,41,42], due to the hyperphagic role of this receptor, and the possible exploitation of its pharmacological blockade, as recently reviewed [43]. It should be recalled that rimonabant, a CB_1_R antagonist/inverse agonist [44], entered the European mass market, showing weight loss benefits but it was soon withdrawn due to the significant side effects [45]. Here, we focused mainly on the role of type-1 Cannabinoid Receptor gene (*CNR1*) gene, which encodes for CB_1_R, and its regulation in food intake and eating behaviors.

## 2. *CNR1* Gene

CB_1_R is one of the most abundant seven transmembrane G protein-coupled receptor of the class A [46]. It is prominently expressed in the central nervous system (CNS) [47] and has attracted great attention as a modulator of different brain functions including appetite, fear, anxiety and pain [48,49,50]. The ECS, as a whole, is comprised of (1) the endocannabinoids (eCBs) anandamide (*N*-arachidonoyl-ethanolamine) and 2-arachidonoylglycerol, which are physiological ligands for cannabinoid and non-cannabinoid receptors; (2) the cannabinoid receptors and non-cannabinoid receptors, such as transient receptor potential vanilloid 1 channels [51,52]; and (3) enzymes responsible for the biosynthesis and hydrolysis of eCBs. Biosynthetic routes are mediated by *N*-acylphosphatidylethanolamines-specific phospholipase D, diacylglycerol lipase, phosphoinositide-specific PLC and *lyso*-PLC, while termination of eCB signaling is terminated through the action of purported transmembrane transporters, followed by hydrolysis by fatty acid amide hydrolase (FAAH) and monoacylglycerol lipase [50].

**Figure 1:**
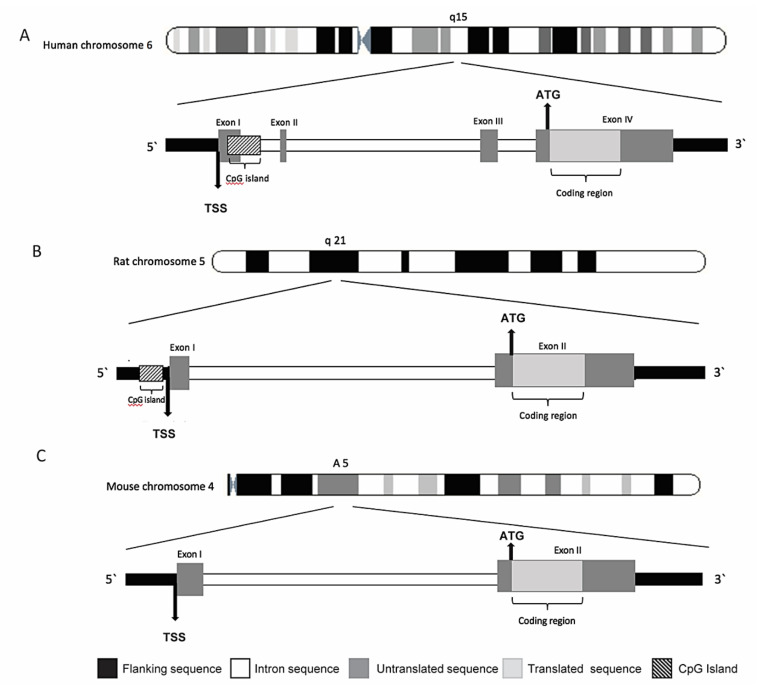
Schematic representation of human(**A**), rat (**B**) and mouse (**C**) *CNR1* gene, with their cromosomial locations.

CB_1_R was first cloned in 1990 and was immediately recognized as the receptor responsible for the effects of marijuana on CNS; it was also reported to be more responsive to psychoactive than non-psychoactive cannabinoids [53]. CB_1_R is encoded by *CNR1* gene, and consists of 472 amino acids in humans, and 473 amino acids in rats and mice, with 97–99% amino acid sequence identity among them [54] (Figure 1). *CNR1* gene is located on human chromosome 6q14–15 [55] and its gene sequence is composed of four exons, with exon 4 containing the entire protein coding region.

Also, in mice and rats the coding region of *CNR1* is contained within a single exon. However, the 5′ untranslated region (5′-UTR) and promoter structures differ between mice and humans [56,57], and these structures are not described in rats [58]. Alternative splicing of portions outside the coding region yields six different 5′-UTR splicing variants. In addition, it appears that multiple transcription starting sites exist within the first 60 base pairs (bp) of the first exon [59]. Three *CNR1* coding region variants for CB_1_R protein isoforms have been identified in humans and non-human primates: (1) the intronless 472 amino acid-long protein, the one known as CB_1_R, (2) the 411 amino acid-long protein, marked as CB_1_Ra and (3) the 439 amino acid-long protein, marked as CB_1_Rb [60,61,62]. Some evidence indicated that CB_1_Ra may also be expressed in the rat brain [59]. Several natural polymorphisms of the human gene have been identified, associated with different responsiveness to cannabinoids [57,63,64,65,66,67]. Alternative splice variants have also been reported, including the canonical long form expressed predominantly in the brain and skeletal muscle and two isoforms with shorter N-terminus, one of which is highly expressed in the liver and pancreatic islet cells where it is involved in metabolic processes [62,68,69]. CB_1_R displays conserved spatial distribution in the CNS among different mammalian species [70]. In the brain, the majority of CB_1_R expressing cells are neurons. In the cortex and in the hippocampus high CB_1_R expressing cells are GABAergic neurons, whereas glutamatergic principal neurons express CB_1_Rs on a lower level [71], while glial cells and astrocytes exhibit only the marginal expression [72,73]. Localization of CB_1_R in the brain, correlated with its role in the control of motor function, analgesia, cognition and memory, is abundant in the cortex, hippocampus, basal ganglia nuclei and cerebellum [46,74,75,76,77]. CB_1_R is also expressed in peripheral tissues like heart, uterus, testis, liver and small intestine, as well as in immune cells [78,79,80] and adipose tissue [81]. In a model of insulin resistance, *CNR1* gene was identified as one of the genes with the greatest increase in expression in adipose tissue [82].

### 2.1. CNR1 Gene in the Control of Energy Homeostasis and Obesity

Circuits in the HYP regulate appetite and energy homeostasis [83] and a key role is played by hypothalamic CB_1_R signaling intertwined with the pathways of metabolic hormones. In fact, for instance, the reduced hypothalamic eCB levels are associated with appetite suppression by leptin [26], while the increased hypothalamic eCB levels are correlated with orexigenic actions of ghrelin, with the involvement of the activation of AMP-activated protein kinase and the inhibition of paraventricular neurons [84].

Using mice lacking *CNR1* gene, it has been documented that eCBs actions on food intake and body weight depend on the functional expression and activity of CB_1_R [85]. In this work, Cota and colleagues demonstrated that germline deletion of *CNR1* in male mice resulted in a phenotype characterized by decreased body weight, reduced fat mass, and hypophagia. Moreover, the study highlighted that *CNR1* mRNA is co-expressed in the HYP with neuropeptides known to modulate food intake [85].

A significantly reduction in body weight was also reported in mice, where *CNR1* gene expression was selectively deleted in the HYP, after 9 weeks of viral-mediated deletion. This effect, without any changes in food intake, suggested an increase in energy expenditure [86]. Further, adult mice, in which *CNR1* gene was deleted in adipocytes, resulted to be protected from diet-induced obesity and associated with metabolic alterations [87].

Again, conditional mutant mice, with *CNR1* deletion in forebrain and sympathetic neurons, known to control energy balance, are resistant to diet-induced obesity and display a lean phenotype [88].

Moreover, the relevant role of CB_1_R in the initiation of milk suckling in pups has been observed [89,90] and, in particular, *CNR1*-knock-out (KO) newborns did not ingest milk on the first day of life, significantly affecting their survival rate [90].

Furthermore, central dysregulation of *CNR1* gene expression has been documented in animal models of obesity in different brain areas, implicated in both homeostatic and hedonic aspects of eating [91,92,93].

In particular, the exposure to a palatable diet resulted in tissue and sex-specific changes in the gene expression of both CB_1_R and type-2 cannabinoid receptor (CB_2_R) in the HYP of offspring and adults. These results clearly indicate that the maternal diet has long-term effects on the development of pups through multiple alterations of signaling homeostatic pathways that include cannabinoid receptors [93].

Gamelin and colleagues (2016) found in the hippocampus of rats, fed with High Fat Diet (HFD), an increase in the *CNR1* mRNA expression compared to rats fed with standard diet. The up-regulation of hippocampal *CNR1* expression was increased with exercise training combined with HFD. Indeed, chronic exercise did not appear to counteract ECS overactivation and, in fact, seems even to induce this effect independently from diet. Moreover, the authors showed that *CNR1* expression in the HYP is not affected by HFD in rats [91].

It was also reported, in rats exposed to HFD, the reduction in CB_1_R binding sites in extrahypothalamic brain regions and CB_1_R density was related to the intake of palatable food, whereas no changes have been observed in the HYP [94]. This does not exclude transient changes in CB_1_R levels or *CNR1* expression over time. Indeed, a transient increase in mouse hypothalamic CB_1_R density, after 3 weeks of HFD, was normalized at the end of the 20 weeks of HFD, suggesting a temporal CB_1_R alteration during the development of obesity [95]. A temporal transcriptional regulation of *CNR1* gene was also proved in the HYP of rats exposed to diet-induced obesity. The analysis of ECS components gene expression revealed a significant and selective increase in *CNR1* mRNA levels at the beginning of obesity development (5 weeks on HFD) as well as after 21 weeks of exposure, when the phenotype was already well-established. Moreover, a consistent selective and significant reduction in DNA methylation at specific Cytosine–phosphate–Guanine (CpG) sites of *CNR1* gene promoter in overweight rats was observed just after 5 weeks, but not 21 weeks on HFD [92].

In the same study, the DNA methylation status of *CNR1* gene was assessed in peripheral blood mononuclear cells from a subset of obese human subjects. An age-based stratification of DNA methylation levels showed a significant reduction of the epigenetic hallmark at *CNR1* promoter in younger (<30 years old) humans with obesity, when compared to age-matching controls. These findings suggest that the regulation of *CNR1* gene is altered mainly at early life stage of phenotype development [92].

Considering other epigenetic modifications possibly occurring in the development of obesity, recently a hypothalamic increase in histone acetylation was reported at *CNR1* gene promoter and was linked to increased receptor expression [96]. Almeida and colleagues hypothesized that maternal fat enriched diet would up-regulate *CNR1* mRNA levels in the HYP of the male offspring at birth [96].

These latter findings support the relevance of environment and lifestyle in the facilitation of diseases progression, including obesity, by engaging epigenetic mechanisms [97], and in meantime could represent an innovative field to produce new strategies of intervention.

Genetic studies have identified several polymorphisms at different locations across the *CNR1* gene that have been associated with obesity and related phenotypes, such as metabolic syndrome and dyslipidemia [98,99,100,101,102,103,104,105,106].

Among others, particular attention has been focused on a silent intragenic biallelic polymorphism in codon 435 of *CNR1* gene, substitution of G to A at nucleotide position 1359 (1359 G/A rs1049353) [107]. This Single Nucleotide Polymorphism (SNP) was reported to be associated with abdominal adiposity [108], Body Mass Index (BMI) [109], intermuscular fat mass [110], and longitudinal changes from healthy to metabolic syndrome occurrence [111]. However, the literature has been inconsistent with respect to *CNR1* polymorphisms and obesity-related markers, with many studies not finding any relevant association with *CNR1* gene variants [112,113,114].

### 2.2. CNR1 Gene in Eating Disorders

EDs, defined in the Diagnostic and Statistical Manual of Mental Disorders (DSM)-V [115], represent a group of conditions characterized by abnormal appetite and eating patterns, accompanied with other physiological as well as psychological disturbances.

The principal mechanisms implicated in the etiology of EDs involve dysregulation of neuronal circuits regulating homeostatic and hedonic aspects of food intake, thus including the ECS signaling.

Candidate gene association studies revealed the association of ECS genes SNPs in EDs. Specific genetic variants of *CNR1*, again rs1049353, as well as FAAH genes were identified in individuals with Anorexia Nervosa (AN) and Bulimia Nervosa (BN) [116], even if an earlier study failed to find associations of the same SNPs in a different AN population [114]. Although *CNR1* rs1049353 is synonymous or silent, thus, not altering the amino acid sequence of the protein, Monteleone et al. suggested that it might have functional effects by changing mRNA stability or translation as already proposed for other SNPs [117]. Moreover, rs1049353 was found associated with lower BMI with unexplained heterogeneity within the human cohort [107].

A microsatellite polymorphism, namely an AAT (adenine-adenine-thymine) trinucleotide short tandem repeat (AAT)n, is present at *CNR1* gene downstream the translation site [57]. It is known that microsatellites might affect transcription efficacy in some genes [118]. This AAT trinucleotide repeat has been found to be associated with restricting and bingeing/purging AN [119].

*CNR1* KO mice display significant body weight loss under standard diet, a resistance to the obesogenic effects of the HFD and a reduced food intake on both diet regimens [120] further supporting the specific association of *CNR1* gene with hypophagia [121,122]. Furthermore, preclinical studies in the Activity-based anorexia (ABA) rat model, found a reduced density of CB_1_Rs in lateral HYP and dental gyrus of the hippocampus [123] and, consistently, it was recently reported the reduction in *CNR1* gene expression in HYP as well as NAc in ABA rats [124]. It has been suggested that the decrease in CB_1_R density might be driven by the decrease in eCBs that are necessary for receptor expression [125]. However, others reported increased CB_1_R availability in ABA rats [126], as well as in AN [127,128] and BN patients [128]. Gerard et al. suggested that in AN, this might act as compensatory mechanisms to chronically hypoactive ECS. Interestingly, short-term starvation increased hypothalamic 2-arachidonoylglycerol concentration in animals [129], whereas a long-term food-restriction (12 days protocol in mice) resulted in whole brain decrease of 2-arachidonoylglycerol [130]. There might occur adaptive strategies for coping with short- and long-lasting food deprivation, as elevated eCB levels might be beneficial to promote food seeking behavior in short term, while down-regulation of this orexigenic signal and reduction of appetite and motivation to eat may aid survival in the conditions of prolonged starvation [131]. Thus, the down-regulation of *CNR1* gene expression might be a compensation for a purported reduced sensitivity of the receptor or a physiological consequence of up-regulated eCBs in these disorders [132]. Following to that, a recent study reported that CB_1_R availability was inversely associated with BMI in homeostatic brain regions of HYP and brainstem both in ED patients and healthy controls, while in the mesolimbic reward system (amygdala, insula, midbrain, striatum, and orbitofrontal cortex), negative correlation was found only in EDs patients [133]. The ECS deviations in homeostatic brain regions most likely present compensatory mechanism aimed at restoring energy balance, while alterations in brain areas implicated in motivation and reward may reflect disordered hedonic eating behavior observed in AN patients.

Endocannabinoids are also implicated in psychiatric comorbidities common in AN, such as anxiety and depression. Chronic stress, anxiety and depression exhibit CB_1_R deficiency and reduced CB_1_R-mediated signaling [134,135], while blockade or genetic deletion of CB_1_Rs has anxiogenic properties [136]. Furthermore, depression in human patients has been linked with several polymorphisms in the *CNR1* gene [117,137,138].

The environment, both independently and in interaction with heritable factors, plays a relevant role in the onset of EDs and may influence gene expression via epigenetic mechanisms [139].

A possible transcriptional regulation of *CNR1* gene, through DNA methylation of its promoter, was investigated in two animal models of AN (one behavioral and one genetic), in order to gain insight on players involved in AN onset and development [124].

More specifically, as an environmental model, it has been used the ABA model, through which rats are exposed to a restricted feeding schedule combined with physical activity, by giving them free access to a running wheel; a “combo” able to induce to a reduction in food intake, dramatic body weight loss and hyperactivity [140,141,142].

The major outcome of the above-mentioned study is that, among genes of the ECS, the expression of only *CNR1* gene resulted to be altered in the ABA group and selectively in the HYP and in the NAc. Moreover, epigenetic analysis on the *CNR1* gene promoter showed a consistent and significant increase of DNA methylation in the NAc; whereas, no changes of the epigenetic mark occurred at the earliest time-point (3 days induction) in the same area, nor in the HYP at neither time-points. Moreover, a significant correlation between body weight and both *CNR1* expression and DNA methylation was reported. No changes were instead observed in the genetic model of AN, the anx/anx mice [143]. This let the authors to suggest that the selective molecular alterations reported in the ABA model were due to environmental cues (i.e., food restriction and physical activity), and not driven by a genetic predisposition.

**Figure 2:**
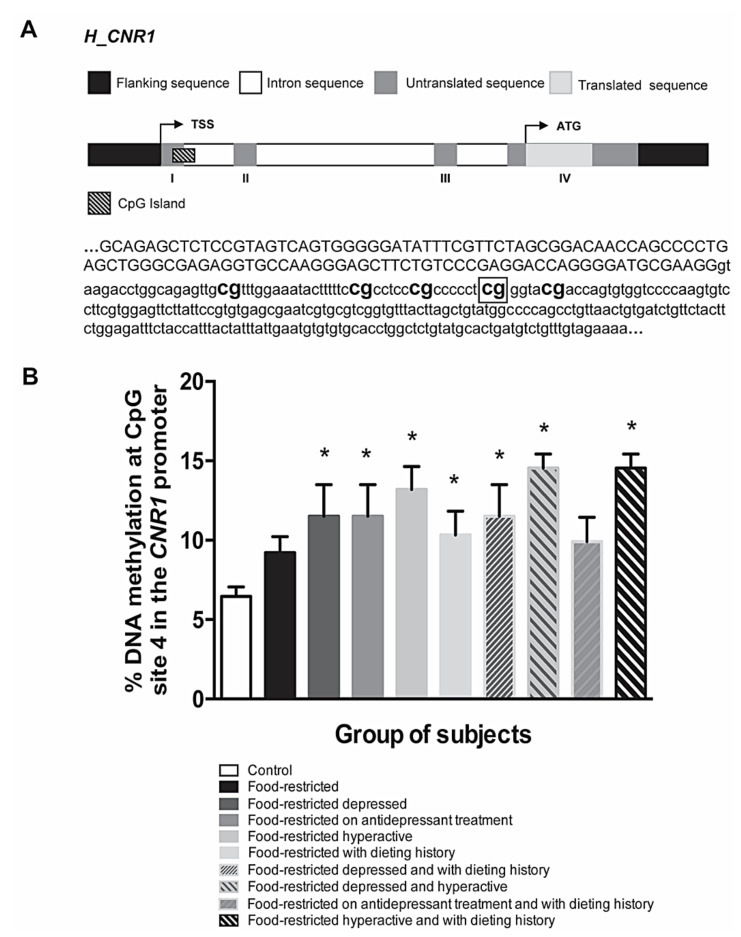
(**A**) Representation of human CNR1 gene and sequence of CpG islands (human GRcH38: crh 6:88165719-88165820) studied for DNA methylation. Flanking sequences are marked in black, UTRs in dark gray, coding regions in light gray, introns in white and CpG islands in lined pattern. Position of ATG, TSS and exons are also reported. Bold text indicates the CpG sites analyzed; framed CG indicates the CpG site number 4. (**B**) DNA methylation status at the CpG site number 4 in the sequence of the human *CNR1* gene under study in food-restricted subjects stratified based on the co-occurrence of different environmental factors. The bars represent the mean of the % of DNA methylation ± the SEM in the different subgroups. Significant differences are indicated as * *p* < 0.05 vs. Control [144].

In this respect, we would like to point out that we recently analyzed the transcriptional regulation of *CNR1* gene in a small population of subjects with restricted eating habits, mainly resembling AN feature [144]. Recruited subjects were females (*n* = 9) selected based on the age (18 to 65 years old, excluding pregnant women and people with known genetic mutations) and BMI (BMI ≥18.5–<25 for normal weight subjects with restricted eating habits, BMI <18.5–≥17 for mild malnutrition, BMI <17–≥16 moderate malnutrition, BMI <16–≥15 severe malnutrition and BMI <15 extreme malnutrition), with healthy subjects (*n* = 21) included as controls. Preliminary findings indicate that, in addition to food restriction, other environmental cues seem to be necessary to alter, in saliva, *CNR1* gene DNA methylation patterns, that we analyzed by pyrosequencing as previously described [92] (see Figure 2A for details of the sequence under investigation). These additional environmental inputs include abnormally high levels of physical activity, previously experienced stressful events and/or dieting history. Among the 5 CpG sites under study at *CNR1* gene, we have observed the most pronounced methylation differences at CpG site 4 (Figure 2B) (unpublished data ref [144]). Moreover, we identified retinoid X receptor alpha (RXR-α) as the transcription factor (TF) that binds this CpG site. Retinoic acid receptors (RAR) form heterodimers with RXR exerting a broad range of biological effects. For instance, RARs are involved in CB_1_R up-regulation in both alcohol- and HFD-induced fatty liver and in mediating CB_1_R expression evoked by eCBs [145]. Moreover, the same CpG site 4 binds another TF, glucocorticoid receptor alpha (GRα), well known for its implication in metabolic conditions of obesity and diabetes and psychiatric illnesses [146]. Agonistic actions on the GR promote fat deposition and central adiposity with adverse metabolic profile, including hyperglycemia, insulin resistance, dyslipidemia and hypertension, observed both in animal models and in human subjects [147,148]. Finally, a synthetic GC was shown to up-regulate peripheral *CNR1* expression, suggesting it is involved in GR-regulated lipolysis making it an attractive drug target in type 2 diabetes and dyslipidemia [149].

Defects in the endocannabinoid signaling, mediated primarily by CB_1_R, have been also implicated in development of binge eating disorder (BED), characterized by recurrent episodes of binge eating, with no compensatory behaviors to prevent weight gain, such as vomiting or laxative abuse [150]; therefore, obese individuals are the most commonly affected by BED [151]. CB_1_R antagonist/inverse agonist rimonabant has been demonstrated to decrease binge eating behavior in female rats by reducing the consumption of the HFD binged, with the accompanying significant body weight loss [152]. It has been also recently demonstrated that female rats under dietary-induced binge eating show a modified central eCB tone in several brain areas within the mesocorticolimbic system, which is the principal neural pathway that drives hedonic eating, as well as reduced CB_1_R density in the prefrontal cortex, probably related to the development and maintenance of this behavior [153]. Moreover, CB_1_R-dependent positive reinforcement appears responsible for maintenance of excessive food intake upon withdrawal [154]. With regards to genetic variants, specific allele has been associated with bingeing/purging AN, but not restricting subtype of AN [115], while several polymorphisms in *CNR1* gene as well as FAAH gene have been associated with addiction and binge-drinking [155,156,157,158]. Recently, the study of ECS components transcriptional regulation [159] in a rat model of binge-like eating showed altered levels of just FAAH gene in the HYP of binge-eating group. BED also has a complex multifactorial etiology, with both genetic and environmental factors implicated [160]. Evidence on the epigenetic of *CNR1* gene in BED are scarce: one report showed a reduced DNA methylation in *CNR1* gene promoter in the prefrontal cortex of eating addicted-like animals, correlated with an elevated expression of CB_1_R protein in the same brain region [161]. More recently, a crucial role of glutamatergic *CNR1* gene has been proposed as part of the regulatory mechanisms of relevance in the loss of inhibitory control for palatable food seeking and consumption [162].

## 3. Conclusions

The ECS is a constitutive signaling system that plays a critical role in energy homeostasis by promoting consumption of palatable food, stimulating fat mass expansion and inhibiting energy expenditure and thermogenesis. Via CB_1_R, eCBs modulate homeostatic and rewarding neural circuitries, and regulates consequently eating behaviors and energy balance, according to food availability: activation of eCB signaling is favorable when access to food is restricted, whereas it promotes obesity and metabolic diseases when food is abundant. Engagement of ECS occurs in conjunction with other metabolic signals, particularly leptin, that act synergistically through their specific neuronal pathways to maintain body energy homeostasis. We provided an overview of the role of *CNR1* gene in EDs and obesity, in order to further stimulate the challenging idea that the modulation of *CNR1* gene transcriptional regulation might represent a promising approach to prevent or to treat these pathologies, in addition to existing pharmacological interventions on CB_1_R [103].

## Data Availability

The data presented in this study reference [144] are available on request from the corresponding author. The data are not publicly available since they are part of an unpublished work.

## Abbreviations

HYP	HYPothalamus
EDs	Eating Disorder
NAc	Nucleus Accumbens
ECS	Endocannabinoid System
CB_1_R	Type-1 Cannabinoid Receptor
*CNR1*	Type-1 Cannabinoid Receptor gene
CNS	Central Nervous System
eCBs	endogenous Cannabinoids
FAAH	Fatty Acid Amide Hydrolase
5′-UTR	5′-Untranslated Region
CpG	Cytosine-phosphate-Guanine
TSS	Transcription Start Site
ATG	Translation start site
bp	base pairs
KO	Knock-Out
CB_2_R	Type-2 Cannabinoid Receptor
HFD	High Fat Diet
SNP	Single Nucleotide Polymorphism
BMI	Body Mass Index
DSM	Diagnostic and Statistical Manual of Mental Disorders
AN	Anorexia Nervosa
BN	Bulimia Nervosa
AAT	Adenine-Adenine-Thymine
ABA	Activity-Based Anorexia
RXR	Retinoid X Receptor
TF	Transcription Factor
RAR	Retinoic Acid Receptors
GR	Glucocorticoid Receptor
BED	Binge Eating Disorder
